# Trends in Personalized Therapies in Oncology: The (Venture) Capitalist’s Perspective

**DOI:** 10.3390/jpm2010015

**Published:** 2012-03-07

**Authors:** Roman Fleck, Daniel Bach

**Affiliations:** Index Ventures, Rue de Jargonnant 2, 1207 Geneva, Switzerland; E-Mail: daniel.bach@indexventures.com

**Keywords:** global industry trends, collaboration BioPharma–Academia, oncology drug development, translational research

## Abstract

Oncology is one of the most important fields of personalized medicine as a majority of efforts in this field have recently centered on targeted cancer drug development. New tools are continuously being developed that promise to make cancer treatment more efficacious while causing fewer side effects. Like most industries, the biopharmaceutical industry is also following certain global trends and these are analyzed in this article. As academia and industry are mutually dependent on each other, researchers in the field should be aware of those trends and the immediate consequences for their research. It is important for the future of this field that there is a healthy relationship among all interested parties as the challenges of personalized medicine are becoming ever more complex.

## 1. Introduction

For the pharmaceutical/biotech industry working on the development of oncology drugs, 2011 was a great year. By the end of December, six novel products were licensed by the Food and Drug Administration (FDA) with most of the approvals issued even before their respective PDUFA (Prescription Drug User Fee Act) dates [1]. Remarkably, two of the six new drugs can be labeled as personalized cancer therapeutics where the companion diagnostic was approved at the same time as the related drug molecule (see Table 1). As we see the pace of oncology drug approvals accelerating, the industry will regards this as a much needed shot in the arm to regain somewhat lost confidence. 

**Table 1:** New cancer drug approvals in 2011 (source: Food and Drug Administration (FDA)).

Molecule	Target	Biomarker	Indication
vemurafenib	BRAF kinase	BRAF V600E mutant	melanoma
vandetanib	VEGFR, EGFR, RETR	*not included on the label*	thyroid cancer
abiraterone	CYP 17A1	*not included on the label*	prostate cancer
ipilimumab	CTLA-4	*not included on the label*	melanoma
brentuximab vedotin	CD30 (ACD technology)	*not included on the label*	lymphoma
crizotinib	ALK	ALK-EML4 fusion protein	lung cancer

BRAF: Serine/threonine-protein kinase B-Raf; VEGFR: Vascular endothelial growth factor receptor; EGFR:epidermal growth factor receptor; RETR: RET proto-oncogen receptor; CYP 17A1: cytochrome P450 17A1; CTLA-4: Cytotoxic T-Lymphocyte Antigen 4; CD30: Cluster of Differentiation 30; ADC: Antibody Conjugated Drug; ALK-EML4: anaplastic lymphoma kinase (ALK); echinoderm microtubule-associated protein-like 4 (EML4) fusion.

Nevertheless, despite the hopes and promises after having deciphered the human genome and despite the tremendous resources being spent on personalized cancer medicines, thus far there are only a handful of drugs on the market that could be labeled as “personalized” (see Table 2) [2]. What we have learned over the last ten years is that cancer is a much more complex disease than we previously thought. For one, each patient is different from every other patient in clinical presentation, prognosis, tumor response and tolerance to treatment in addition to differences in risk of recurrence, second malignancy and long-term complications of treatment. And yet, only recently have we begun to understand the heterogeneity in the genetic make-up of human cancer [3] and the inter-individual variation of the human genome [4] that enables a more personalized approach to cancer treatment. However, as the price of sequencing technologies has dramatically dropped where the sequencing of a full human genome now costs only a few thousand dollars instead of millions, the analysis of the cancer genome and examination of variation in germline DNA will become a much easier task to tackle. Together with the advances in epigenetics, drug metabolism, and post-translational regulation of cancer proteins, we should be in a much better position now than ever before to match the treatment to the tumor characteristics and select the optimal drug and drug dosage for each patient. 

**Table 2:** Approved cancer drugs that can be used with biomarker test.

Molecule	Target	Biomarker	Indication
busulfan ^ab^	DNA	Ph+	CML
irinotecan ^ab^	Topoisomerase I	*UGT1A1	colorectal cancer
cetuximab ^ab^	EGFR	EGFR+, KRAS-wt ^c^	colorectal cancer; head and neck cancer
imatinib ^ab^	bcr-abl (c-kit, PDGFR)	Ph+, C-Kit+	CML, GIST
transtuzumab ^ab^	ErbB2	ErbB2 overexpression ^c^	breast cancer; gastrointestinal cancer
getifinib ^b^	EGFR	EGFR-TK mutant ^c^	NSCLC
tamoxifen ^ab^	ER	ER+	breast cancer
denileukin diftitox ^a^	IL2R	CD25+ ^c^	cutaneous T-cell lymphoma
mercaptopurine ^ab^	DNA synthesis	*TPMT	leukemia; non-Hodgkin’s lymphoma
dasatinib ^b^	bcr-abl, src	Ph+	ALL, CML
thioguanine ^ab^	DNA	*TPMT	acute leukemia, CLL
erlotinib ^b^	EGFR	EGFR+	NSCLC; pancreatic cancer
nilotinib ^b^	bcr-abl (and others)	Ph+	CML
arsenic trioxide ^b^	apoptotic	PML/RAR alpha gene+	AML
lapatinib ^ab^	EGFR, ErbB2	HER-2+ ^c^	breast cancer
panitumumab ^ab^	EGFR	EGFR+, KRAS-wt ^c^	colorectal cancer
capecitabine ^ab^	DNA synthesis	*DPD	breast cancer, colorectal cancer
aromatase inhibitors ^abd^	estrogen synthesis	*Aromatase+, ER+	breast cancer
DNA intercalators ^abe^	DNA	*Topoisomerase IIα copy number	breast cancer

^a^ Food and Drug Administration (FDA) approved; ^b^ European Medicines Agency (EMA) approved; ^c^ Biomarker test is included in the drug label; ^d^ This category includes several drugs as Letrozole, Anastrozole, *etc*; ^e^ This category includes several drugs as Epirubicin, Doxorubicin, *etc.* TPMT: thiopurine methyltransferase; UGT1A1: Uridine diphosphate glucuronosyl-transferase 1 family, polypeptide A1; DPD: dihydropyrimidine dehydrogenase; EGFR:epidermal growth factor receptor; HER-2: human epidermal growth factor receptor-2; bcr-abl/Ph = Philadelphia chromosome; ER: estrogen receptor; PDGFR: Platelet-derived growth factor receptors; ALL: acute lymphoblastic leukemia; AML: acute myeloid leukemia; CLL: Chronic lymphocytic leukemia; CML = chronic myeloid leukemia; GIST: gastrointestinal stromal tumors; NSCLC: Non-small-cell lung carcinoma. (*): these tests are rarely used, but have been important at some point during the drug development or in clinical practice.

## 2. Global Trends in Oncology Drug Development

**Figure 1:**
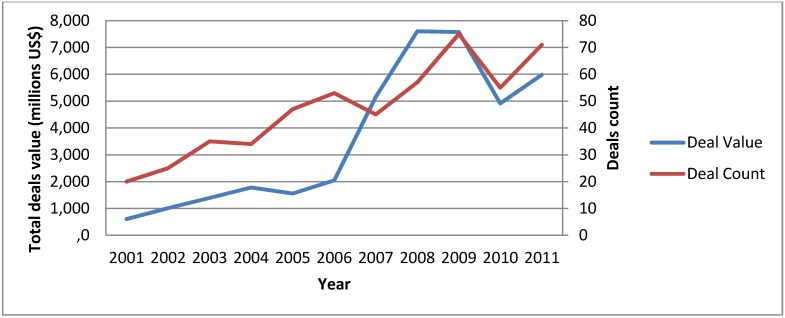
Mergers & Acquisition (M&A) activity in Oncology. Numbers of deals signed with molecules in Preclinical or Phase I are depicted, as well as total cumulative deals value (Source: Medtrack).

Oncology remains a very attractive therapeutic area for biopharmaceutical companies where the number of drugs in clinical development more than doubled between 2000 and 2010. In particular, it was the early stage pipelines that grew disproportionally, indicating that basic research continues to be translated into clinical drug development [5]. In the same period, the number of deals concerning oncology drugs in early clinical stage and the dollars invested in those deals has steadily increased, making oncology the most active therapeutic area in the M&A (Mergers & Acquisition) space (see Figure 1). With this in mind, we identified several trends at biopharmaceutical companies which are at the forefront of shaping the future of personalized cancer medicines:

### 2.1. Progressive Shift towards Molecularly Targeted Therapies

An analysis of Pharma’s oncology pipelines reveals that there is a pronounced shift towards more targeted therapies, away from indiscriminate cytotoxic agents or broad cell cycle inhibitors. While in 2000 only a quarter of the molecules in clinical development could be considered targeted (including cell surface receptor binders, kinase inhibitors, growth factor inhibitors, and angiogenesis inhibitors), that ratio had increased to two-thirds by 2010. And this ratio would be even greater if line extensions (such as reformulations of already approved molecules, ‘me-too’ or ‘me-better’ drugs) were excluded from consideration and only novel agents considered [5]. A side effect of the more specific nature of the target is an increased competition between several companies as they often choose to go after the same mode of action, as evidenced by the six EFGR inhibitors in clinical development or the five BCR-ABL tyrosine kinase inhibitors [5,6]. While it may represent a low risk strategy for the drug developers to target already validated mode of actions and while it also ensures that the most optimized molecule for a particular target will reach the market, we may ask ourselves whether more efforts should be devoted to finding a more diversified set of targets. 

Another trend in targeted therapies is trying to match it to patients earlier in treatment. When imatinib (Gleevec) was first introduced for chronic myeloid leukemia (CML), it was tested in chronic phase CML patients, a late stage of the disease where cancers become heterogeneous and particularly resistant to treatment, and the response rate was less than 15 percent [7,8,9]. Despite this modest response rate imatinib was eventually approved in 2001 in an unprecedented 11 weeks, the fastest FDA review period of any cancer drug at that time. In contrast, when tested in the front line setting, and with the introduction of similar but increasingly potent second- and third-generation drugs, patients’ response rate is now reaching 90 percent, and their expected survival is more than 25 years [10,11]. Despite these obvious successes it is still an uphill battle with the regulatory agencies to test novel oncology agents in early stage patients. It certainly poses an ethical dilemma for the agencies as patients may lose valuable treatment time on best standard of care if the experimental treatment proves to be ineffective. Nevertheless, it is certainly difficult to speculate but we may have discarded many useful agents because they did not work in late stage patients whereas they could have been very useful in a frontline setting.

It will be crucial for the success of these therapies to benefit from a more flexible and adaptive approach to clinical trial design and many such efforts at the agencies are under way to come up with a regulatory framework.

### 2.2. Combining Targeted Therapies

In June of 2009, Merck and AstraZeneca announced a paradigm shift in oncology drug development where the two companies would collaborate to research a novel combination anticancer regimen composed of two investigational compounds targeting different kinases, MK-2206 from Merck (an AKT inhibitor) and selumetinib from AstraZeneca (a MEK-1 inhibitor) (see Table 3). In preclinical experiments the combination of these two molecules had shown strong synergies well beyond additive effects [12]. This was the first time that two large pharmaceutical companies had established a collaboration to evaluate the potential for combining candidate molecules at such an early stage of development as both molecules had just finished Phase I clinical trials. Up to then, such combinations would have only been studied if one or both of the drugs had entered late-stage development or received marketing approval. It was a bold move as figuring out the correct dosing regimen for a novel molecule targeting a new mode of action is already challenging, doing so with two new such molecules simply potentiates the challenge. And it was certainly no accident that two large Pharma companies with a long history of cancer drug development partnered to share resources and experience to meet this challenge. In the meantime, other companies have followed suit, such as Merck Serono collaborating with Sanofi on combining two kinase inhibitors (Sanofi’s SAR245408, a PI3K inhibitor, in combination with Merck Serono’s pimasertib, a MEK-1 inhibitor) or Roche collaborating with BMS on combining a kinase inhibitor with an immuno-modulatory antibody (Roche’s vemurafenib, a BRAF kinase inhibitor in combination with BMS’s ipilimumab, a CTLA4 inhibitor). 

**Table 3:** Some examples of Pharma–Pharma collaborations in oncology.

Company 1	Company 2	Compound 1	Compound 2	Modality	Indication
Merck & Co.	AstraZeneca	MK-2206 AKT inhibitor	selumetinib MEK-1 inhibitor	combination	lung cancer
Merck Serono	Sanofi	pimasertib MEK-1 inhibitor	SAR245408 PI3K inhibitor	combination	solid tumors
BMS	Roche	ipilimumab CTLA4 inhibitor	vemurafenib BRAF inhibitor	combination	melanoma

### 2.3. Market Fragmentation

Another visible trend is the fragmentation of the oncology market because of an increased focus on “rare tumors” (defined by an incidence rate of less than 3 pts./100,000/year) as well as a sub-segmentation of the more common tumors (such as lung, breast, colorectal, gastric and prostate) into more specific diseases based on their genetic make-up. Ten years ago, almost two-thirds of the oncology pipelines were filled with molecules indiscriminately targeting (meaning, not directed towards a particular subset of patients) the big five cancers. By now, this percentage has dropped to about half with a further drop expected in the next five years. This trend not only results from a better understanding of the underlying tumor biology but also from the implementation of a new business model thinking at Pharma where small patient numbers in a particular disease do not necessarily mean small profits. As Genzyme has demonstrated in the rare/orphan disease space, it is feasible to ask for premium prices if a given therapy is highly effective albeit in a small patient population. A personalization of therapy usually means targeting a smaller subset of patients that share a particular phenotype but then we expect higher efficacy and/or lower toxicity in this population. A more effective and better tolerated therapy can command higher prices that would allow Pharma to recoup its investment in the development of the molecule. A slow recognition within Pharma of the economics of a personalized approach to cancer care is settling in and will fuel further development. 

### 2.4. Use of Biomarkers as Companion Diagnostics

The use of biomarkers to identify patients who are most likely to respond or be resistant to a given therapy is at the core of personalized cancer care and will likely continue to increase. This practice has coined the term companion diagnostic, to describe those biomarker-based tests that will assist the physician in making a treatment decision by selecting a drug that is associated with this particular test. The opportunities for biomarker-directed drug development are exciting, offering the potential to limit enrolment of candidates for clinical studies to those most likely to benefit from the treatment under study. This enables the design of studies that demonstrate larger effects, but with smaller numbers of patients and hopefully a faster path towards regulatory approval. Overall, the economics to get a drug from inception through the approval process should be greatly improved. And certainly it would be the hope of the Pharma developer if there were other indications in which this drug could be useful, and which can then be explored post approval of the primary indication. A prime example for this development path is imatinib, which was originally developed for CML but now has been approved in four other indications (including gastrointestinal stromal tumors (GIST), *etc.*) [13]. Originally targeting a small niche indication, imatinib has grown to a multi-billion dollar franchise by now. 

However, we should not lose sight that there are still significant obstacles and risks associated with the widespread use of biomarkers in clinical development. First, we need to recognize that understanding the underlying cancer biology and identifying suitable molecular targets and biomarkers is still very challenging. Target/pathway mutations, up-regulation of target, or parallel pathway activation provide a challenging mix to find the right biomarker. And as we have seen for example with cetuximab (Erbitux, a monoclonal antibody directed against epidermal growth factor receptor, EGFR) and EGFR expression, an invalid biomarker (overexpression instead of KRAS mutation) can lead to confounding clinical trial design and results [14,15]. Similarly, a technically difficult or wrong assay type can provide inconclusive or unreliable results. Therefore, confidence in the biological relevance of the marker and extensive analytical validation of biomarker assays are required to move biomarkers into broader use. 

Second, the required skills to develop a companion diagnostic test are very different from the skills needed to develop a drug; and usually the two skill sets do not reside within the same organization (with a few exceptions). Drug developers are well aware of this and rather than developing those skills internally, they prefer to partner with companies specialized in diagnostic test development early on in order to optimize the process. Historical barriers to collaboration such as the ownership of information and intellectual property, profit sharing, and others have been resolved to the best interest of both sides. In some case, these alliances do not only cover a single drug-diagnostic development program, but even a much broader horizontal collaboration. A recent example is the alliance between Dako and AstraZeneca, where the tissue-based diagnostics developer will broadly collaborate with the pharmaceutical company on the development of multiple biologics and small molecule drugs. This is only one example in what is expected to be a long series of strategic alliances between diagnostic companies and Pharma to expand their personalized medicine efforts (see Table 4) [16].

**Table 4:** Some Pharma-Diagnostic Companies collaborations. In some cases these are broad research collaborations aimed to develop not yet disclosed drugs and/or diagnostic tests (Source: Business Insight Ltd.).

Drug developer	Dx developer	Drug	Diagnostic	Indication
Amgen	DxS	panitumumab	K-Ras	colorectal cancer
Eli Lilly	Genomic Health	cetuximab	mRNA signature	colorectal cancer
Ipsen	BioMérieux	*several n.d.*	*several n.d.*	breast cancer
Merk KGaA	OncoMethylome	cilengitide	MGMT gene methylation	brain cancer
Pfizer	Genomic Health	*several n.d.*	mRNA signature	renal cancer
OSI	Dako	ertolinitib hydrochloride	EGFR	NSCLC
Merck & Co	OncoMethylome	temozolomide	MGMT gene methylation	brain cancer
AstraZeneca	Dako	*several n.d.*	*several n.d.*	*several*

*n.d.: not disclosed.*

Finally, and to make matters even more challenging for drug developers, they are also facing significant regulatory hurdles to developing biomarker assays for clinical use as both the test and the drug must meet regulatory standards for marketing approval and clinical use. As the review of diagnostic test and drug is located within different divisions of the agency, different review processes and approval standards are applied, thus making it hard to come up with clinical trial designs that satisfy both divisions. It is clear that a streamlining of these processes is essential for a more rapid drug development and fortunately, some initiatives at the agencies are under way that will hopefully bear fruit soon.

### 2.5. Move towards Proof of Relevance *versus* Proof of Concept

A lot of terrific science is being explored in academic labs around the world, but it is actually the biopharmaceutical industry with its financial muscle that ultimately decides which pathway and related therapy is progressed into human testing and ultimately, if successful, to market approval. Researchers will need to understand what makes a particular drug or pathway more attractive than others in order to draw the investment needed to progress their approach into further development. A clear differentiation strategy showing ’Proof of Relevance’ (PoR), and not just ’Proof of Concept’ (PoC), will become more and more important. In this context, we define relevance as a strategy to develop a drug which will be clearly differentiated in the market place compared to other drugs against the same disease.

A lot of important research and drug development is carried out in universities and smaller biotech companies which have become a feeder for the pipelines of large Pharma companies. As most of these outfits are not well enough capitalized to conduct later stage clinical trials it has been fairly common practice to progress a drug development program up to PoC trials (typically Phase IIa trial) before hoping to partner or license the molecule to a larger organization with deeper pockets. A PoC trial in oncology usually involves the comparison of a new therapy on top of best standard of care (BSC) *versus* BSC alone. As third party payers become increasingly reluctant to pay tens of thousands of dollars for a new therapy with marginal benefits, it will be important to show that the new therapy is not only slightly better than BSC (and thus potentially meriting regulatory approval) but rather much better; be it in terms of efficacy, tolerability, relapse rate, patient convenience, or costs. And it will be important to benchmark the new therapy against molecules that will (likely) be approved within the next couple of years. Only such a clear differentiation strategy will ensure that if the new therapy will ever make it to market it will also provide the commercial success needed in order for the developer to recoup its investment. And thus, molecules that can show this “proof of relevance” will be able to attract the financial resources from a Pharma partner or further investments from the financial community. 

A recent example where this PoR strategy has worked extremely well is the development of Plexxikon’s vemurafinib in melanoma. Already early on, Plexxikon could show in preclinical animal experiments that vemurafinib was able to induce partial or complete tumor regression and improved survival, without associated toxicities. In the same models, BSC showed only marginal benefits. The data looked so compelling that the Swiss Pharma company Roche decided to partner this molecule when it was still in Phase 1. Roche was the ideal partner as it had the capability to develop a companion diagnostic for vemurafinib in-house, which greatly aided in patient group stratification in later stage trials. On the back of strong data and an efficient clinical development strategy, vemurafinib was recently approved in a record five years after the investigational new drug (IND) filing. The value of this drug was then further recognized by Daiichi Sankyo’s acquisition of Plexxikon for a record setting $805M upfront and $130M in milestone payments. 

## 3. How Venture Capital Is Deployed in Personalized Medicine

Noting the above developments, the venture capital community’s approach to investing in personalized medicine in particular and in life science projects in general, has changed over the last five to seven years. It will be important for investigators interested in starting a company to understand how this impacts the structure of an “investable” company and what they can do to make their project more attractive for venture capital. While most of these trends are quite general in nature, they certainly will apply to personalized medicine drugs as well, an area which is of great importance to the venture capital community. 

### 3.1. The Asset-Centric *versus* Company-Building Approach

In a typical company-building model, several assets (or molecules) are pooled under the same roof to diversify the risk of one asset failing. Simply put, even if the lead asset fails, resources can be immediately diverted to the other assets thus ensuring the further existence of the company. As it turns out, investors tend to focus the main part of their due diligence on the lead molecule and also assign most of the company’s value to it. As a result, there tends to be less stringency for selecting the second, third, *etc*., molecule and hence their likelihood of success is even lower. Nevertheless, in case the lead molecule fails, money and resources will be dedicated to those assets in a desperate attempt to keep the company afloat and its workers employed. More often than not though, this way of building a company has led to failure. Just to be clear, many successful biotech companies were built according to this model and may continue to be built. But even in the successful cases, this company model rarely provided investors in these companies with sustainable returns. It’s easy to see how a lot of money was ill-spent in our industry and why the financial community is becoming increasingly reluctant to make commitments to the life science sector.

Another way of building a biotech company is to use a platform technology as its foundation. The promise of any platform is that it can generate multiple drug candidates with unique features in a short period of time, thus making it “easy” to kill projects and move on to the next. The idea then is to quickly move a candidate molecule into clinical development, gaining clinical validation on the lead molecule, and thus “de-risking” the whole platform (and making it more valuable). But as it is common practice in our industry, the value of a company is mainly driven by the value of its most advanced product rather than the platform technology. As a consequence, investors are rarely rewarded for the development and validation of the technology platform, which in our experience can cost anywhere between $30M to $50M. Therefore, it is not surprising that investors are more and more unwilling to finance technology platforms. Nevertheless, we do need new platform technologies and we do need to find ways to finance them. One potential way to approach this is to early on attract larger partners who are willing to share the development cost in return for a limited access to the platform or individual products. For the platform company this is a double-win as such a deal not only provides financial resources but also validation of its science by an external partner. Going forward, this is probably the preferred way to finance platform companies in the long run.

Learning its lessons from the failures of the company build-out model, the venture capital community has increasingly embraced the so-called “asset-centric” model. It discards the idea of having back-up molecules or a platform behind those molecules and calls for one molecule per company. The infrastructure of the company is kept to a bare minimum with most of its activities being outsourced to professional service providers. Only a small core team needs to oversee the most strategic parts of the drug development process (e.g., clinical trial planning & execution, regulatory, CMC development, *etc*.). These teams tend to be entrepreneurial minded, professional drug developers who work closely with the scientific founders on the drug development plans. The idea of this model is that a very ruthless discipline can be applied where drug candidates can be discontinued at any point in time without considerations of consequences for company or its employees, something big Pharma companies often find hard to do. To the contrary, management of these companies is even incentivized to abandon ill-fated projects as their financial upside is also tied to the success of a molecule, thus working on a molecule with no future makes no sense. Of course, a team that has worked well on a given project, even if the molecule did not succeed, will be asked to work on the next molecule/company, something that is never in short supply at a good venture capital company. But it needs to be said that the asset-centric approach may not be compatible with all different kinds of molecules, and careful consideration has to be given to each individual case.

### 3.2. The ‘New Mode of Action’ *versus* ‘Fast Follower’

We find two distinct types of molecules that are being developed in the biopharmaceutical world. There are those molecules that are based on new biology and thus are directed against a largely unknown target involving a completely ‘new mode of action’ (MoA). By contrast, we consider as ‘fast followers’ molecules where the MoA has been validated by drugs that are either in late stage trials or already on the market. Fast followers are usually differentiated either by higher potency or better PK/PD properties compared to the lead drug and can include either a new drug substance or the same substance as the lead drug but where the delivery modality was improved.

It is not difficult to see that the risk profiles of these two types of molecules are completely different. The new MoA will need a lot of validation in preclinical experiments as well as safety studies to provide enough comfort to move it into the clinic. It will be key to understand whether biology that is operational in study animals will also translate into man. So that means that there are two major risk factors for the development: the new biology as well as the safety of the molecule. The fast-follower molecule, by contrast, does not share the new MoA risk as the biology is usually well understood and validated in man. However, the safety of the fast-follower needs to be established as well be it because it is a new molecular entity or a new delivery method. Another risk factor stems from the uncertainty over whether the fast follower provides for enough differentiation from the lead drug at the bedside to make it a commercial success. Venture capitalists carefully assess the different risk profiles of the new MoA *versus* fast follower molecules when setting up a new company, as the financial structure may be quite different.

### 3.3. Why Personalized Medicine Is “Hot” for Venture Capitalists

One of the key words when venture capitalists make an investment decision is “differentiation”, meaning whether the technology or drug will make a difference for the patient at the end of the long development path. Even if a given drug will make it to approval, commercial success will only be guaranteed if there is enough of a difference felt by the patient in order to make a switch form his/her current medication to the new drug. Differentiation can be quite broadly defined, be it better efficacy, less side effects, or more convenient dosing (e.g., longer intervals) compared to the best standard of care. Thus without credible scientific evidence that the new investigational drug can be differentiated along those lines it will be difficult to find a serious investor to finance the development of, for example, yet another statin drug, no matter how big the market is. A potential key to differentiation can be provided by personalized medicine, which makes it therefore attractive for venture capital. Personalized medicine provides the possibility to develop a companion diagnostic that can identify a target population in which a particular drug has a better chance of working. It is an often cited fallacy that a drug that addresses a smaller population is less attractive. To the contrary, a drug that works in fewer people but shows a tremendous benefit over standard of care will be able to command premium prices (as we have seen with drugs for rare diseases) and thus attractive returns for investors. Also, the likelihood of regulatory success may be greater as the tolerance for side effects is greater if the treatment benefit justifies it. Moreover, the clinical development of personalized drugs is typically cheaper and faster, because it requires smaller clinical trials in a more homogeneous patient population, hopefully yielding clearer and more robust results.

Personalized medicine is also profitable for payers and the healthcare systems as it may ensure that only those patients who are most likely to respond will be prescribed a given drug. Indeed, treating non-responding patients provide an unnecessary drain of money and resources to the already stressed healthcare systems not to mention needless suffering due to side effects. Any drug that can reduce the direct cost to the healthcare system will eventually have a bigger chance of commercial success. 

In general, we prefer to invest in a molecule addressing a target which is selectively found only in a subpopulation of breast cancer patients compared to a molecule addressing the general breast cancer population, despite it being a bigger market. Personalized medicine can provide great tools to “de-risk” a project early on, something which is very appealing to potential investors.

## 4. What the Venture Capital/Biopharmaceutical Industry Is Interested in

Over the last ten years we have made some great inroads on the vision of personalized medicines and it is to be expected that the pace of progress will accelerate over the next ten years. But as the financial pressure on funding pharmaceutical projects will only increase we need to be more careful in the future on what we spend our resources. It is clear that in the past, a lot of resources were spent on ill-fated, pie-in-the-sky projects and we may not have this luxury anymore in going forward. Thus it will be crucial to understand where we spend our valuable resources to maximize the output. A prudent mix of scientific focus, innovative business models, and increased collaboration should help us to make great progress. 

### 4.1. A Selective Look at MoA’s Currently in Early/Late Stage Trials

**Figure 2:**
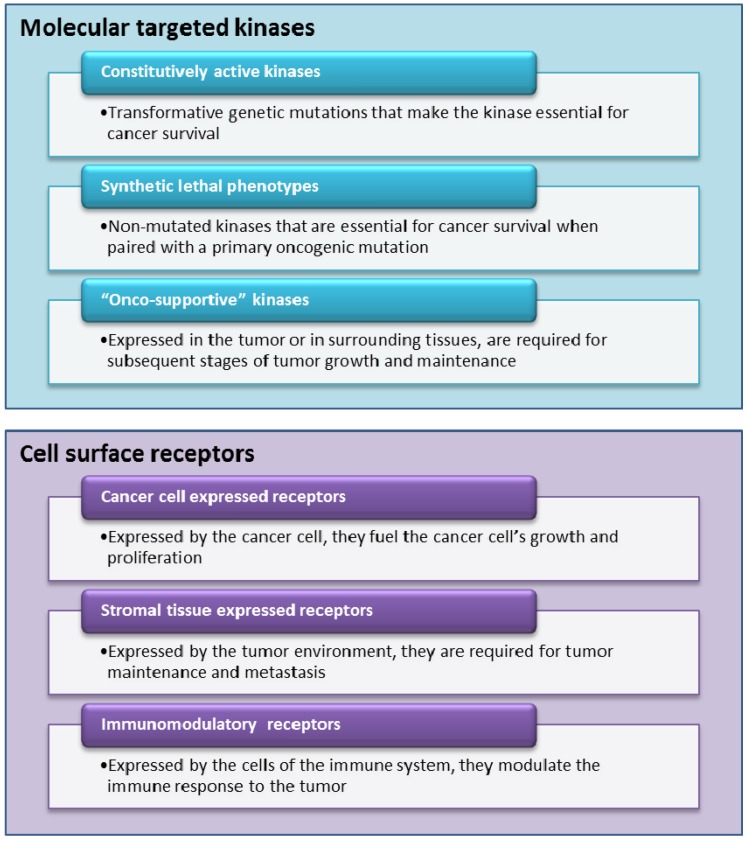
Selective look at ‘new mode of action’ (MoA) currently in development.

Most of the drugs currently in development for personalized oncology fall into two classes, either molecularly targeted kinase inhibitors or ligands for cell surface receptors (see Figure 2). Typically, kinase inhibitors are small molecules while cell surface receptors are mainly targeted by monoclonal antibodies. We decided to not discuss a third class, therapeutic cancer vaccines, as many of the big pharmaceutical companies (with some notable exceptions) are still in wait and see mode. Despite the approval of sipuleucel-T (Provenge) and all the excitement it has provided for the field, we need to see more real successes before significant resources will be invested in this approach.

#### 4.1.1. Molecularly Targeted Kinases Inhibitors

With more than 500 distinct kinases identified and over 60 kinase inhibitors in clinical trials or FDA approved [5,6], this important class of proteins is a rich area for therapeutic drug development. It is beyond the scope of this paper to review all kinase targets that are currently being exploited for drug discovery but there are some distinct trends that can be distilled. 

There are three distinct classes among kinases that can be identified. First, there are kinase targets that escape normal regulatory mechanisms following genetic mutation or translocation. The constitutive activity of this class of kinase target, also called oncogene addiction, makes them essential for survival and/or proliferation of the cancer cell and thus susceptible to the appropriate kinase inhibitor. One of the most notable successes in this area was the discovery of the V600E mutation, located in the activation loop of BRAF [17]. The targeted inhibition of this particular mutation has led to the discovery and subsequent approval of vemurafenib in melanoma. For a second class of kinase target, inhibition of the kinase results in a synthetic lethal phenotype when paired with another non-lethal mutation in the particular pathology of the tumor cell. Although rarely mutated in cancer, these kinases are preferentially required for the survival and/or proliferation of cancer cells and may be located in key signaling pathways downstream of transforming oncogenes. Examples include mitogen-activated protein kinase kinase 1 (MEK1) and 2 (MEK2), which are located in the critical mitogen-activated protein kinase (MAPK) pathway, mammalian target of rapamycin(mTOR), which is located in the phosphatidylinositol 3-kinases (PI3K) protein kinase B (Akt) signaling system, and the ribosomal S6 kinase (RSK) [18]. While there are no synthetically lethal kinase inhibitors in the clinic, this concept of synthetic lethality has thus far been exploited with the anticipated utility of inhibitors of poly-ADP(ribose) polymerase (PARP) in BRCA1/2 deficient breast cancers [19,20] A third class of kinase targets are expressed in the tumor or in surrounding tissues and are required for different stages of tumor formation and maintenance in the human host. Notable examples are the vascular endothelial growth factor (VEGFR) and fibroblast growth factor receptor (FGFR) kinases [21], which are important in developing and sustaining tumor blood supply, as well as the pyruvate kinase which is required for the tumorigenic switch to aerobic glycolysis. 

With the recent approvals of vemurafenib (targeting the V600E mutation in the serine/threonine-protein kinase B-Raf (BRAF)) and crizotinib (targeting the anaplastic lymphoma kinase (ALK) and echinoderm microtubule-associated protein-like 4 (EML4), ALK–EML4 fusion protein), both of which fall into the first category of kinase inhibitors, this class became the poster child of personalized medicine in cancer and we will hopefully find and define similar such targets (e.g., inhibitors of the V617F mutation in Janus kinase 2 (JAK-2) or of the D816V mutation in the proto-oncogene c-Kit (cKIT) are currently under clinical investigation) [8]. As these kinds of mutations are readily identified by DNA sequencing they lend themselves to easier engineering of mechanistically relevant cellular assays that can be used during the course of inhibitor discovery and optimization. One problem that is inherent to this class of inhibitors is the duration of action as escape mutations can develop very readily [22,23]. Even on this front we make progress with the recent approval of nilotinib that targets mutations that develop after imatinib therapy [24] (and another such molecule, ponatinib, is in Phase II clinical trials [25]). It is to be expected that there will be a lot of emphasis by drug developers on this class of molecules.

Kinase targets of the second and third classes are not directly transforming but instead are required for the survival, proliferation and/or tumor genesis of cancer cells. As such, they can be highly context dependent and much more difficult to investigate in preclinical experiments. Especially the mechanistic basis for selective cytotoxicity of many inhibitors in the second class is yet poorly understood. Typically, kinases that can be targeted through a synthetic-lethal interaction specific to cancer cells must be investigated and validated by a largely empirical process. Thus we may only be able to “personalize” these kinds of targets in large post approval studies which hopefully will allow us to identify genetic patient phenotypes that would reap the most benefit. Likewise, kinase targets required for tumor formation and maintenance are usually evaluated in animal tumor models that are suboptimal reflections of the disease evolution in humans, so better tumor models are needed to increase the success rate of these targets. Thus we can expect a much slower pace of development in these kinds of target classes.

#### 4.1.2. Ligands for Cell Surface Receptors

Cell surface receptors provide an almost equally rich class of targets. Unlike kinases, which reside inside the cell, they are expressed on the cell surface where they are activated by other protein ligands, thus setting a signaling cascade in motion. Since protein–protein interactions are notoriously difficult to disrupt by small molecules the majority of the cell surface receptors are being targeted by monoclonal antibodies. 

Also, in this class we can broadly distinguish between three classes of targets. First, there are growth factor receptors which fuel the cancer cells’ growth and proliferation. The most famous example and probably the first example of a targeted therapy for which there was a companion diagnostic available is trastuzumab (Herceptin), a monoclonal antibody which binds to the human epidermal growth factor 2 receptor (HER2) [26]. The *HER2* gene is amplified and overexpressed in 15–25% of breast cancers, and HER2-positive carcinomas are typically associated with high tumor grade and invasiveness. However, despite having made a large impact on treating HER-2 positive patients, the majority of cancers that initially respond to Herceptin begin to progress again within one year [27]. Other examples in this class include epidermal growth factor receptor (EGFR), insulin-like growth factor receptor 1 (IGF-1R), hepatocyte growth factor receptor (HGFR), platelet-derived growth factor receptor (PDGFR), *etc.* Cell surface targets in the second class are not necessarily directly expressed by the tumor cell but rather by the host environment feeding the tumor and are required for tumor maintenance and metastasis. The most famous example for this class is bevacizumab (Avastin) a monoclonal antibody that is approved for the treatment of non-small cell lung cancer, metastatic breast, colorectal, and kidney cancer. Bevacizumab binds to vascular endothelial growth factor (VEGF) and prevents it from interacting with receptors on endothelial cells, blocking a step that is necessary for the initiation of new blood vessel growth (angiogenesis) [28]. Despite its commercial success bevacizumab has also serious side effects (e.g., serious bleeding events) and its approval in breast cancer has been recently revoked by the FDA (despite EMA’s decision to keep this indication on bevacizumab’s label). Other examples in this class include placental growth factor (PGF), fibroblast growth factor receptor (FGFR), and inhibitors of many different kinds of integrin receptors (α_v,2,4_β_1–7_). The third class can be broadly summarized as immunomodulatory agents which do not directly interact with the tumor but stimulate the immune system to attack malignant cells. The most prominent example here is ipilimumab, a monoclonal antibody against cytotoxic T-lymphocyte protein 4, CTLA4, and which was recently approved for the treatment of late stage melanoma [29]. CTLA4 is an inhibitory protein expressed on T-cells and blocking it is part of a strategy to inhibit immune system tolerance to tumors. As this strategy can be of general use for many tumor types, ipilimumab is currently tested in other cancer indications. Other targets in this class include CD19/20, IL-2R, CD40, CD56, *etc.*

It is more difficult to personalize therapies targeting cell surface receptors as most of these targets are expressed on normal cells as well. And since all of the pathways that are stimulated by growth factors are redundant/overlapping to a certain degree, interfering with one may only yield a modest effect. This could be the reason why we see resistance against trastuzumab in HER-2 positive tumors occurring so rapidly as back-up pathways may take over and continue to provide the positive signal for tumor growth. Thus it may be necessary to target more than one growth factor receptor simultaneously in order to choke off tumor growth signals. With the advent of protein engineering there is now an exciting array of multifunctional fusion proteins that can target two or three of these receptors simultaneously and some of them are already being explored in clinical trials. Hopefully, in the not too far future we will know whether this strategy will enhance the therapeutic benefit. 

By definition, agents that target the host tumor environment and angiogenesis cannot be personalized as these targets are not transformed and thus their expression level, genetic make-up, *etc.* should be the same for every patient. Nevertheless, each individual cancer may spark a somewhat different microenvironment and we need to understand how to best characterize this environment and how it responds to these agents. Thus a personalization strategy then is more directed towards how we use the agents we already have in hand (and there are a few more in clinical trials) based on some yet to be defined biomarkers, and how we can incorporate them in an individualized treatment regimen to maximize their therapeutic effects.

With the immunotherapy approaches, the key challenge we face is that despite signs of them working, the therapeutic effect thus far has been fairly modest. We need to better define how they are working, how we can amplify the efficacy, and which patients will benefit the most from them. In this regard, retrospective identification of predictive biomarkers is likely to be an important strategy well after approval of the drug. Once identified, the predictive power of the biomarker can then be explored in a hypothesis driven prospective clinical trial.

### 4.2. From Bench to Bedside and Back to Bench

With the advent of personalized medicine, we are progressing from a population-based empirical ‘one drug fits all’ paradigm to a focused approach where rational companion diagnostics support the drug’s clinical utility by identifying the most responsive patient sub-group. But to date, most of the handful of predictive biomarkers that have successfully gained utility as companion diagnostics were identified through retrospective analysis of clinical trial data and coincidental *ad hoc* genetic analysis. The challenge now is to exploit current techniques that enable predictive biomarkers to be identified in a systematic prospectively-driven fashion, allowing drug development to progress hand-in-hand with the associated biomarker, and thereby open up a few more hypothesis-based approaches to developing personalized cancer therapy. We may find two types of predictive biomarkers useful, those that tell us about drug resistance and those that identify drug responsiveness by a tumor. While the identification of the latter biomarkers is more attractive for drug developers, we must not lose sight that the former is equally important. Funding agencies should step in more vigorously and sponsor trials that would allow us to identify patients for which a particular treatment would not be useful, thus saving time, unnecessary suffering and costs for the patient.

We also need more investments to develop biomarkers not only for decision making in clinical trials, but also for hypothesis generating clinical studies that could lead to the identification of new biomarkers, targets and resistance mechanisms. Translational medicine is no longer a one-way process from ‘bench to bedside’, but rather a continuous dynamic and iterative cycle between lab and clinic, where clinical results are not only proving (or disproving) the hypothesis that was generated in the lab but also providing feedback to the lab for further hypothesis generation. Furthermore, the timescales for iterative cycles between the laboratory and clinic have to be shortened, necessitating a highly integrated multidisciplinary and team-based approach to drug development. This has to be enhanced by collaborations between academic centers, the pharmaceutical industry and also regulatory bodies. 

### 4.3. Molecular Profiling with Whole Genome Sequencing

A recent study at MD Anderson Cancer Center showed that matching targeted therapies to the tumor’s specific gene mutation could improve the response rate by five-fold compared to patients who were treated with non-matched therapies. In this heterogeneous, heavily pre-treated patient population the median overall survival was 13.4 months for the matched group *versus* 9 months for the unmatched group [30]. At this point we can test tumors for up to 12 gene mutations but as the rate of technology is rapidly advancing we may see up to 100 of those mutations being identified and validated in the near future. However, conventional sequencing of each of these genes for clinical practice would be cost prohibitive and would not identify mutations in novel cancer associated genes. For example, the current cost to comprehensively sequence the breast cancer susceptibility proteins BRCA1 and BRCA2 alone is approximately $4,000. An alternative is emerging with the advent of whole-genome sequencing, which has at least two key advantages over candidate gene sequencing. For one, whole genome sequencing provides a comprehensive and nonbiased approach to mutation detection. Its strength is that it is a generic and stable platform for detection of mutations, and no special approaches are required for specific diagnostic settings. And the cost of whole genome human genome sequencing is falling rapidly, with a current cost estimate below $10,000 per genome. If we apply Moore’s law (in this case, a cutting of costs in half every 18 months), we can expect the price of whole genome sequencing to be affordable for the routine clinical setting within the next five to seven years. Also, the sequencing timeline should greatly speed up and we can expect that clinical-grade whole-genome sequencing could soon be possible within four weeks of sample collection, making it clinically relevant also for tumors where quick initiation of therapy is required. Secondly, whole-genome sequencing can detect structural variants (such as deletions, amplification, inversions, and translocations) that are often overlooked by conventional sequencing. All classes of mutations can be detected in an entirely unbiased fashion, allowing for confirmation of a suspected diagnosis, even if caused by a rare or unusual mutation.

Nevertheless, it is clear that we will not have a specific drug in our arsenal for each individual genetic mutation we detect and whose relevance is often not fully understood. But we may find ourselves often surprised to discover that a particular drug may work through a very different mode of action than we originally assumed. Molecular profiling technologies will give us better tools for such an analysis and we may find that an existing drug can address genetic mutations for which it was not specifically developed. It will be pertinent as we accumulate more whole genome sequencing knowledge and subsequent treatment outcomes, that this knowledge will be publicly available to the greatest extend. Along this line, Dana Farber Cancer Institute in collaboration with Brigham & Women’s Hospital just announced the launch of a massive study to test cancer patients’ tumors for hundreds of genetic aberrations in an effort to build a more comprehensive understanding of the underpinnings of cancer and how to tailor patients’ treatment.

The biotech community also took note of this trend, for example, with the founding of Cleave Biosciences, which this year attracted the largest round of start-up financing, $42 million, from a group of blue-chip venture capital groups. This start-up will focus on pathways in protein homeostasis to match its targeted drugs to a subset of patients whose tumors are regulated by the same pathways using molecular profiling technologies. Cleave assembled a team of drug developers with a long history of targeted drug development.

Beyond the study of the whole genome, transcriptome, proteome and metabolome, systems biology aims to comprehensively study all markers configuring the signature of disease and its evolution [31]. The wide-spread availability of high-throughput technologies allows us to visualize up- or down-regulation of thousands of molecules in parallel, modifications in their activity, localization, and interactions between them. The dynamic study of these processes will hopefully allow us to understand how the cancerous cell and the tumor will behave in response to drug therapy. This complex network of interactions defines a unique fingerprint for each patient and their disease and will be at the heart of future personalized medicine. 

### 4.4. Novel Models of Collaboration and Partnerships

As personalized oncology turns out be an ever more challenging task, collaboration between all players (academics, biotech and Pharma companies) will be essential to turn this vision into a reality. Early and translational research collaborations among drug developing companies or between drug developing companies and academic centers have often been fraught with complications, as haggling over ownership, intellectual property, and development costs have hampered the free flow of information necessary to develop complicated development programs. New ways of thinking and open innovation business models will be required to bring personalized oncology to the bedside. 

One idea that has recently gained momentum is collaboration at a “pre-competitive” level where drug discovery tools are being developed by a consortium of Pharma companies and those tools, once mature, can be freely exploited by members of the consortium (e.g., Enlight Biosciences). Another idea may come from the information technologies (IT) world where we have learned that collaborative open innovation did not destroy the business model of IT firms but rather expanded their markets. As was discussed earlier, there have been some interesting efforts to increase collaboration where novel molecules from different companies have been jointly developed as combination products. The next step would be to increase collaborative efforts at the discovery stage, which is at the heart of innovation and where collaboration would probably yield the greatest results (e.g., the efforts of Pfizer to establish collaborations in discovery genomics). This kind of open collaboration has been perceived as difficult though, as ownership—and thus future profits—are defined at the discovery stage. But just as IT companies let others play with their code, why don’t Pharma companies let others play, free of charge, with patents or projects in which they have no current interest (e.g., by posting them in a freely accessible database)? It is not hard to imagine that a biotech company may come up with new approaches that turn a stalled project into a successful one. What the Pharma company might get in exchange is the right to data (*i.e.*, new knowledge) and a right of first refusal or negotiation. This could provide the desired win–win situation for all stakeholders, including patients.

## 5. Conclusions

It is clear that the field of personalized medicine has seen great progress in the last couple of years, with most activity in the field of oncology. What we have learned thus far is that most cancers are very heterogeneous and that we need to reclassify these diseases on a molecular level and not just on broad phenotypes. As such we will need to focus on the efficacy of our drugs in carefully characterized patients and not in broad patient populations. 

Based on the duration of action from our targeted therapies it emerges that it is unlikely that there is a single magic bullet that can address a particular cancer in the long run and we need to think about how we can tackle tumors from different angles. Multi-targeted approaches, be it combinations of targeted molecules or engineered multifunctional proteins, will play a key role. Also, we will need to majorly innovate on how we design our clinical trials and demonstrate clinical efficacy. More flexible and adaptive trial designs that will also feedback to our preclinical research will lead the future. 

At this point we have developed some companion diagnostics that tell us whether an associated drug will be efficacious. The future will lie in a personalized molecular characterization that will allow us to design the appropriate therapeutic combination for each patient and at each moment of the disease progression. Molecular profiling technologies are the centerpiece to this idea and with the advent of ever cheaper sequencing technologies as well as the advance of systems biology, it should be feasible to make personalized medicine part of standard clinical practice in the not too distant future. And despite the potentially increased cost of adding molecular profiling diagnostics to the regular clinical practice, the avoidance of unnecessary treatments will pay for this many times over and benefit patients and payers alike. Finally, we must not lose sight that the increasing complexity of personalized drug development requires a new approach and thinking about how we approach research collaborations and how we do business tomorrow. 
